# Intrinsic hippocampal connectivity is associated with individual differences in retrospective duration processing

**DOI:** 10.1007/s00429-023-02612-3

**Published:** 2023-01-25

**Authors:** Alice Teghil, Alessia Bonavita, Federica Procida, Federico Giove, Maddalena Boccia

**Affiliations:** 1 Department of Psychology, “Sapienza” University of Rome, Via dei Marsi 78, 00185 Rome, Italy; 2 Cognitive and Motor Rehabilitation and Neuroimaging Unit, IRCCS Fondazione Santa Lucia, Rome, Italy; 3 PhD Program in Behavioral Neuroscience, Sapienza University of Rome, Rome, Italy; 4 MARBILab, Museo Storico della Fisica e Centro Studi e Ricerche Enrico Fermi, 00184 Rome, Italy

**Keywords:** Resting-state fMRI, Time perception, Timing, Hippocampus, Medial temporal lobe

## Abstract

The estimation of incidentally encoded durations of time intervals (retrospective duration processing) is thought to rely on the retrieval of contextual information associated with a sequence of events, automatically encoded in medial temporal lobe regions. “Time cells” have been described in the hippocampus (HC), encoding the temporal progression of events and their duration. However, whether the HC supports explicit retrospective duration judgments in humans, and which neural dynamics are involved, is still poorly understood. Here we used resting-state fMRI to test the relation between variations in intrinsic connectivity patterns of the HC, and individual differences in retrospective duration processing, assessed using a novel task involving the presentation of ecological stimuli. Results showed that retrospective duration discrimination performance predicted variations in the intrinsic connectivity of the bilateral HC with the right precentral gyrus; follow-up exploratory analyses suggested a role of the CA1 and CA4/DG subfields in driving the observed pattern. Findings provide insights on neural networks associated with implicit processing of durations in the second range.

## Introduction

The representation of the duration of events provides the basis to structure internal and external inputs, and is a key prerequisite to high-order cognitive processes, including future thinking and autobiographical memory (Ivry and Schlerf 2008; Matthews and Meck 2016). A crucial distinction in the study of duration processing concerns whether elapsing time is explicitly attended to (prospective duration processing), or time becomes the focus of attention only after it has elapsed (retrospective duration processing) (Zakay and Block 2004). Over the last decades, several studies have investigated brain mechanisms supporting the prospective processing of durations. At the neuronal level, prospective time processing has been shown to involve spatiotemporal patterns of population activity, that define specific neural trajectories (Tsao et al. 2022), and can be identified in multiple brain regions, including the striatum, premotor and medial frontal cortex (Paton and Buonomano 2018; Wang et al. 2018; Zhou et al. 2020). Accordingly, at the system level, explicit prospective duration judgments have been mainly associated with the activity of a distributed network, that involves primary sensory, motor and premotor regions, as well as the inferior frontal and posterior parietal cortex, and the striatum (Merchant et al. 2013; Coull and Droit-Volet 2018). Within this network, regions such as the inferior frontal and posterior parietal cortex appear to be mainly involved in prospective duration judgments in the second range (Lewis and Miall 2003; Wiener et al. 2010). Cognitive models highlight a key role of attentional resources in prospective time processing in the range of seconds (Zakay and Block 2004). It has been thus suggested that the preferential involvement of prefrontal and parietal regions in prospective time processing of second-durations reflects the attentional and working memory demands associated with this process (Coull et al. 2011; Lewis and Miall 2003).

At difference with prospective duration processing paradigms, retrospective ones require participants to estimate already elapsed durations that were not attended to; thus, duration information in this kind of paradigms has to be extracted from memory (MacDonald 2014). Retrospective time processing has thus been crucially linked to episodic memory by cognitive models, and proposed to depend on the retrieval of contextual information associated with a sequence of events, that is automatically encoded in medial temporal lobe regions (Zakay and Block 2004).

Early indication for a role of the medial temporal lobe in the representation of elapsed time has been provided by studies examining the consequences of lesions to the hippocampus (HC) in non-human animals. Rats with a lesion to the hippocampal subfield CA1 appear to be unable to make object-odor associations when the object and the odor are separated by a 10 s delay (Kesner et al. 2005). Moreover, trace fear conditioning, involving a temporal delay between stimuli, has been shown to be impaired by inactivation of the dorsal HC (Raybruck and Lattal 2011; Sellami et al. 2017). Other studies have provided complementary evidence that damage to the HC does not affect prospective duration processing per se, but rather appears to impair memory for durations in prospective paradigms (Meck et al. 1984; see MacDonald 2014, and Lee et al. 2020, for reviews). These findings support the possibility of a key involvement of the HC in extracting duration information from memory, and thus in the retrospective processing of durations (MacDonald 2014; Lee et al. 2020). In recent years, more direct evidence of a mechanism in the medial temporal lobe that could represent time incidentally in service of episodic memory, thus possibly supporting retrospective duration processing, has been provided by the discovery that specific neural populations in the HC exhibit a temporal selectivity, firing at consecutive moments within time intervals in the order of seconds (Pastalkova et al. 2008; MacDonald et al. 2011, 2013; Kraus et al. 2013, among others). These “time cells” have been mainly described in hippocampal CA1 subfield in rodents (Pastalkova et al. 2008; MacDonald et al. 2011, 2013; Kraus et al. 2013; Ning et al. 2022), and their firing patterns are proposed to provide an evolving temporal context during the encoding of event sequences (Eichenbaum 2014). Time cells have been very recently described also in the human HC, during sequence learning (Reddy et al. 2021) and an episodic memory task, in which their activity predicted the temporal clustering of memories (Umbach et al. 2020).

The study of brain correlates of retrospective duration processing in humans has been somewhat limited to date. In line with studies in non-human animals, a role of the HC in retrospective time processing has been indirectly suggested by some fMRI studies. Increased pattern similarity in the HC was reported for elements of a sequence judged to be closer in time (Ezzyat & Davachi 2014; Deuker et al. 2016) and pattern similarity in the HC between the study and test phase of a match-mismatch task was affected by changes in the duration of empty intervals between stimuli, also when such durations were processed implicitly (Thavabalasingam et al. 2018). These studies, however, used picture stimuli arranged in sequences devoid of an intrinsic meaning (Ezzyat and Davachi 2014; Thavabalasingam et al. 2018), raising concerns in terms of their ecological validity. In the only study assessing temporal proximity judgments following a more naturalistic learning experience, participants encoded durations during a spatial navigation task, not allowing to decouple temporal from spatial encoding (Deuker et al. 2016). Thus, whether the HC supports explicit retrospective duration judgments in humans, and putative neural dynamics involved, are still poorly understood.

Over the last decades, the study of intrinsic functional connectivity in relation to individual differences in behavior has proven to be particularly suited to investigate brain mechanisms supporting cognition (Stevens and Spreng 2014), and individual differences in cognitive functioning have been associated with inter-individual variations in resting-state connectivity in several domains (among many others, Chong et al. 2017; Deng et al. 2016), including prospective duration processing (Teghil et al. 2020). Thus, to better characterize neural dynamics involved in the retrospective processing of time, and based on the abovementioned findings suggesting a key role of the HC in this process, here we tested the hypothesis that differences in intrinsic connectivity patterns of the HC are associated with individual variations in retrospective duration processing. We assessed retrospective duration processing using a novel task based on ecological stimuli, allowing to overcome the limitations of previous studies.

## Methods

### Participants

Thirty participants took part in the study. 2 participants reported having guessed that the task involved duration judgments (see task description), thus analyses were performed on a final number of 28 (mean age = 26.89, SD = 2.825, 17 females). This sample size is in line with previous studies investigating the relation between individual differences in high-level cognitive processes and resting-state functional connectivity (Chong et al. 2017; Teghil et al. 2020; Deng et al. 2016). All participants were right-handed, had normal or corrected-to-normal vision, and no history of neurological or psychiatric disorders. The study was designed in accordance with the principles of the Declaration of Helsinki and approved by the ethical committee of IRCCS Fondazione Santa Lucia, Rome. Informed consent was obtained from all participants.

### Retrospective duration processing task

**Fig. 1:**
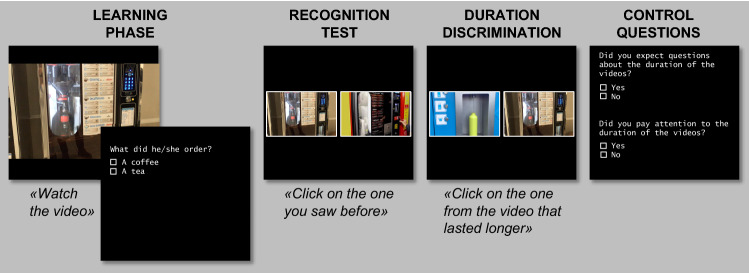
Schematic representation of the duration discrimination (DD) paradigm (only a single trial for each phase is displayed)

Retrospective time processing was assessed using a novel duration discrimination (DD) paradigm, performed outside the scanner. A schematic representation of task events is shown in Fig. 1. During the learning phase, participants were shown 5 video clips displaying everyday activities (e.g. calling the lift, ordering at the vending machine), shot from a first-person perspective. 3 video clips were shot in interior, whereas the last 2 in the exterior; none involved interactions with other people or animals. Video clips were presented in random order. Participants were not told in advance that the task involved time perception, but were simply instructed to carefully watch at the video clips, specifying that questions would have been made on the video clips’ content. The video clips lasted 5, 11, 17, 23 and 29 s. After each video clip, a multiple-choice question (2 possible answers) was presented concerning its detail. A recognition task followed the learning phase: in each trial (*N* = 5), a still frame extracted from one of the 5 video clips was presented together with a distractor, and participants had to choose which of the two images was taken from one of the observed video clips. The side of presentation of the targets and distractors was counterbalanced. Then, in the DD task, participants were shown pairs of still frames coming from the video clips, and asked to decide which of the two corresponding video clips lasted longer (10 trials, corresponding to all pairings of still frames from the 5 video clips). The number of correct responses (maximum; 10) was recorded and used in following analyses. The presentation order of the pairs, along with the side of their presentation, was randomized. Finally, two multiple-choice questions were presented asking participants whether they (1) had guessed that the task involved time processing, and (2) Had paid attention to the duration of the video clips. Participants answering affirmatively to such questions were excluded from analyses. The administration order of the DD paradigm and the MRI session was counterbalanced across participants.

### Image acquisition

MR images were collected using a Siemens MAGNETOM Prisma scanner, operating at 3T, and equipped with a 32-channel head coil. For each participant we acquired two resting-state fMRI scans using a T2٭-weighted gradient-echo echo-planar imaging (EPI) sequence, a multiband factor of 4, and an isotropic voxel size of 2.4 mm^3^ (60 slices, Field of View [FOV] 208 × 208 mm^2^, repetition time [TR] = 1100 ms, echo time [TE] = 30 ms, flip angle = 65°, no in-plane acceleration) (Moeller et al. 2010; Feinberg et al. 2010; Xu et al. 2013). We acquired 300 fMRI volumes in each run, including 4 dummy scans before each run, which were discarded. Two spin-echo EPI volumes with phase encoding in opposite direction, no multiband acceleration and the same geometrical and sampling properties of functional runs were acquired for field mapping (TE = 80 ms, TR = 7000 ms). T1-weighted structural images were acquired for each participant using an MPRAGE sequence (Hess et al. 2011; Tisdall et al. 2012). Volumetric imaging included 176 slices, isotropic resolution 1 mm^3^, TR = 2500 ms, TE = 2 ms, Inversion Time [TI] = 1070 ms, flip angle = 8°. During resting- state fMRI scans, participants were asked to lay at rest with eyes closed and not to fall asleep.

### Analyses and results

Resting-state data were analyzed using the CONN toolbox (v. 20b) (Whitfield-Gabrieli & Nieto-Castanon 2012; http://www.nitrc.org/projects/conn). A field map was computed from the spin-echo EPI images acquired with opposite encoding polarity (Holland et al. 2010). After removal of the first 4 scans, functional images were corrected for head movements and B_0_-distortion, including motion  ×  field interaction (realignment and unwarping, Andersson et al. 2001) using the first volume as reference, and resampled to a voxel size of 2 × 2 × 2 mm^3^. Time series were interpolated to correct for slice-timing distortions. Structural images were segmented in gray matter, white matter (WM), and cerebrospinal fluid (CSF) for successive use during removal of temporal confounding factors, and normalized to MNI space. After normalization, ART-based scrubbing (Power et al 2012) was applied (z-threshold = 5 and movement threshold = 0.9 mm). This led to the removal of 1.918% of data. Functional data were smoothed using a 6-mm^3^ full-width half- maximum (FWHM) Gaussian kernel. Temporal confounding factors (time-courses of WM and CSF BOLD signals, a linear trend, and the six motion parameters derived from the previous realignment procedure) were removed from the BOLD time series of functional data, regressing them out at each voxel. A band-pass filter (0.008–0.09 Hz) was then applied to resulting residual time series. Denoising and band-pass filtering were performed separately for each session, then runs were concatenated and the rest bivariate correlations were estimated jointly across the two runs, according to the standard pipeline implemented in CONN (Whitfield-Gabrieli and Nieto-Castanon 2012).

**Fig. 2:**
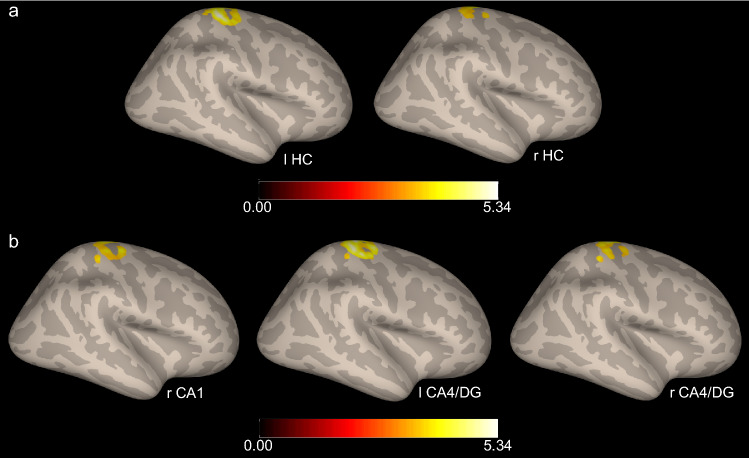
Results of the seed-to-voxel analyses performed on **a** the left (leftmost panel) and right (rightmost panel) HC ROIs, and **b** hippocampal subfields ROIs. For both analyses, duration discrimination accuracy was in the regression model at the second level analysis. *l* left hemisphere, *r* right hemisphere

Analyses of performance in the behavioral paradigm showed that participants discriminated still frames extracted from the video clips with 100% accuracy. Mean accuracy in the DD task was 9.036 (SD = 0.962, range 6–10). We then assessed the relation between DD performance and seed-to-voxel connectivity patterns of the left and right HC. Seeds were selected from the FSL Harvard–Oxford Atlas (Desikan et al. 2006) as implemented in CONN. For each of the two HC seeds, accuracy scores (number of correct responses) of participants in the DD task were entered in the multiple regression model at the second level analysis, using a voxel threshold of *p* < 0.001 uncorrected and a cluster-size p-FWE corrected of *p* < 0.025 (0.05/2) (one-tailed positive). We found a significant association between DD accuracy and individual variations in the functional coupling between the left HC and a cluster of voxels in the right pre/postcentral gyrus (peak at MNI: + 26, − 28, + 64, cluster-size p-FWE = 0.000005) (Fig. 2a, leftmost panel). DD accuracy was also associated with variations in the intrinsic coupling between the right HC and the right precentral gyrus (peak at MNI: + 10, − 28, + 72, cluster-size p-FWE = 0.01623) (Fig. 2a, rightmost panel).

To further specify the contribution of different hippocampal regions to the observed connectivity patterns, in a second exploratory analysis we derived ROIs for the hippocampal subfields from the Cobra Atlas (http://cobralab.ca/atlases/Hippocampus-subfields/) (Winterburn et al. 2013). The following ROIs were derived bilaterally: CA1, subiculum (Sub), CA4/dentate gyrus (CA4/DG), CA2/CA3, for a total number of 8 ROIs. Then, for each ROI, we performed a seed-to-voxel analysis entering accuracy in DD in the multiple regression model at the second level analysis. A voxel threshold of *p* < 0.001 uncorrected and a cluster-size p-FWE corrected of *p* < 0.006 (0.05/8) (one-tailed positive) were used. We found a significant association between DD accuracy and the strength of the functional coupling between the right CA1 and CA4/dentate gyrus and the right precentral gyrus (CA1: peak at MNI + 08, − 32, + 70, cluster-size p-FWE = 0.000157; CA4/DG: peak at MNI + 08, − 30, + 70, cluster-size p-FWE = 0.0003), as well as between DD accuracy and the strength of connectivity of the left CA4/DG with the right pre/postcentral gyrus (peak at MNI: + 28, − 28, + 56, cluster-size p-FWE < 0.001) (Fig. 2b).

## Discussion

Overall, present findings provide evidence of an association between inter-individual variations in intrinsic HC connectivity patterns, and behavioral performance in the retrospective discrimination of durations in the second range. To our knowledge, this is the first report explicitly linking connectivity patterns of the HC to retrospective duration processing. Differences in retrospective duration discrimination were found to be linked with variations in the strength of the functional coupling of the HC with the right pre-/postcentral gyrus. On the one hand, this finding is in line with previous evidence of intrinsic connectivity between the HC and regions of the somatomotor network (Ezama et al. 2021; Seoane et al. 2022), and specifically with studies reporting a functional coupling between the anterior portion of the HC and the right precentral gyrus (Robinson et al. 2015; Boccia et al. 2017). On the other hand, the right precentral gyrus has been previously implicated in duration processing, especially in prospective paradigms. Specifically, lesions to the right precentral gyrus have been associated with lower performance in discriminating durations in the second range (Gooch et al. 2011), and this region is consistently activated during suprasecond, non-motor time processing tasks (Nani et al. 2019). These results parallel findings that neurons in the primary motor cortex display a climbing pattern of activity during delay periods, appearing to encode elapsing time also in absence of movement (Lebedev et al. 2008; Knudsen et al. 2012). It has been proposed that time processing is grounded in action (Coull and Droit-Volet 2018). Supporting this account, the perceived presentation duration of pictures of different body postures has been reported to be affected by their content, suggesting that the reactivation of action dynamics may influence duration judgments (Droit-Volet et al. 2013). Although the functional significance of the involvement of precentral regions in time perception is still unclear, it is possible that the involvement of the primary motor cortex in the perception of time reflects the activation of a mental representation of action (Coull and Droit-Volet 2018). Here we used ecological stimuli, and durations were defined by video clips shot from a first-person perspective. This leaves open the possibility that in line with evidence that episodic autobiographical memory entrails at least in part a sensorimotor simulation of the original experiences (Ianì, 2019), individual differences in estimating the duration of such clips from memory may be associated with variations in the strength of neural dynamics supporting some reactivation of movement features. Although such an interpretation is speculative at this point, this would be consistent with evidence that lesions to the right precentral gyrus are associated with lower performance in tasks tapping body schema (a dynamic representation of body parts derived from motor and sensory inputs that interacts with motor systems during action, Schwoebel and Coslett 2005) (Boccia et al. 2020), and more generally tie well with a key role of the right hemisphere in body representation (Di Vita et al. 2017, 2019).

Notably, it has been suggested that bidirectional interactions may exist between prospective and retrospective timing processes (MacDonald et al. 2014; Tsao et al. 2022). The HC is strongly anatomically connected with the striatum (that is acknowledged to play a key role in prospective time processing within a cortico-thalamic circuit, Merchant et al. 2013, for a review) (Maller et al. 2019), and, in rodents, sequential activity spanning intervals of minutes has been observed in both the striatum and the HC (Shikano et al. 2021). Present findings would be in line with the possibility of an interaction between prospective and retrospective duration processing mechanisms, showing that individual variations in retrospective duration discrimination performance are associated with the functional coupling of the HC with a brain region part of the network supporting prospective time processing (Nani et al. 2019). Since the medial temporal lobe has been proposed to act as a convergence hub during successful context retrieval, interacting with other cortical areas (Watrous et al. 2013), it is possible that these interactions may happen as well with regions supporting prospective timing in the second range. Cognitive and neural dynamics supporting interactions between prospective and retrospective time processing mechanisms, however, are still largely unknown. Further research is thus needed to provide support to this possibility, also considering that data reported here, exploiting individual variations in brain connectivity and behavior to characterize neural networks supporting retrospective time processing, are correlational in nature.

Present results also provide preliminary evidence that the observed connectivity pattern of the HC could be driven by the connectivity of specific hippocampal subfields, namely CA1 and CA4/DG. As mentioned above, several studies reported the presence of time cells in rodents CA1 (Pastalkova et al. 2008; MacDonald et al. 2011; Kraus et al. 2013). Evidence for a role of this subfield in human memory for time has been provided by recent findings that multivoxel patterns of activity in CA1 during recognition and mental replay allow to decode individual sequences composed by different images and temporal delays (Thavabalasingam et al. 2019). The association between CA1 intrinsic connectivity and retrospective duration processing is thus in line with evidence that time cells also exist in humans (Reddy et al. 2021; Umbach et al. 2020), supporting the temporal organization of episodic memories.

The DG has been widely implicated in pattern separation (Duncan and Schlitchting, 2018, for a review), that is in the encoding of associated experiences as distinct neural representations. In the elderly, DG gray matter volume predicts the reduced performance in distinguishing between memories of similar stimuli (Dillon et al. 2017), and representations of highly similar events can be successfully decoded in the DG, but not in other subfields (Berron et al. 2016). The DG has been further proposed to support spatiotemporal context discrimination, since it shows lower pattern similarity for correctly discriminated spatial and temporal distances (Copara et al. 2014).

Preliminary evidence found here for a positive association between resting-state functional connectivity of CA1 and DG and duration discrimination would be further in line with the proposed functional specialization of the HC subfields. Providing an evolving temporal signal, time cells in CA1 are thought to participate in the disambiguation of individual events within a more complex experience (MacDonald et al. 2011). Moreover, pattern separation mechanisms may be involved in discriminating between similar representations of the duration of events, in line with evidence that the DG is sensitive to changes in spatial as well temporal information within sequences (Azab et al. 2014). Present results thus suggest the possibility that higher ability to discriminate the duration of events from memory may be related to stronger intrinsic connectivity patterns of hippocampal subfields which functional specialization may support the retrieval of the temporal context of such events (possibly thanks to the activity of time cells in CA1) and the disambiguation between the overlapping memory representation of events duration. This would be in line with evidence that both CA1 and DG are involved in the temporal separation of events (Hunsaker and Kesner 2013). Further studies assessing connectivity patterns of the HC during an active retrospective duration processing task will be needed to provide stronger support to the hypothesis that CA1 and DG are involved in temporal context retrieval and pattern separation for events within a sequence in the second-minute range.

Some limitations of the present work should be acknowledged. First, we used hippocampal subfields ROIs defined based on an existing atlas (Winterburn et al. 2013), rather than individually defined seeds based on hippocampal segmentation. Although the atlas used in the present study has been shown to be highly reliable (Winterburn et al. 2013), studies using segmentation procedures are needed to provide confirmation to the results concerning hippocampal subfields, and to provide more compelling evidence on their possible functional role in retrospective time processing. Second, recent research on large neuroimaging consortium data increasingly points out to the importance of investigating brain-behavior associations in very large datasets to improve the reproducibility of findings (Marek et al. 2022). Thus, future studies are warranted to replicate present results in larger samples.

It is also worth noting that recent research has highlighted that collecting resting-state fMRI data in eyes-open versus eye-closed conditions may affect the pattern of observed results (e.g. Wei et al. 2018; Agcaoglu et al. 2019), further proposing that these two conditions may, respectively, correspond to an “interoceptive” and “exteroceptive” modes of processing (Xu et al. 2014). Differences have been specifically observed in the auditory, visual and somatomotor networks, with increased connectivity of the visual network in eyes-open conditions and increased connectivity in the auditory and somatomotor networks in eyes-closed conditions (Agcaoglu et al. 2019). Although present results concerning the connectivity between the HC and precentral regions in relation to individual differences in a specific behavioral task are unlikely to be explained by acquisition conditions, this variable should thus be also kept in consideration in studies aiming to replicate the present data.

Finally, in this study participants performed at ceiling in recognizing pictures from the observed video clips. This recognition test was aimed to ensure that participants had actually paid attention to the stimuli. However, including more fine-grained measures of memory for encoded stimuli in future studies may be important to understand the impact of memory on retrospective duration processing, in line with behavioral evidence that recollection and mental replay of events affect duration judgments (Faber and Gennari 2015; Jeunehomme and D’Argembeau, 2019). Pattern similarity in the left HC across single trials has been recently reported to be modulated by the presence of boundaries; also, pattern similarity in the same region predicted duration judgments, with greater pattern change predicting longer subjective duration (Sherman et al. 2022). These findings thus further suggest the importance to investigate how features known to modulate event memories may affect the neural representation of the duration of such events.

To conclude, present results provide initial evidence that intrinsic HC connectivity is associated with individual variations in the ability to discriminate incidentally encoded event durations. Futures studies should further investigate network dynamics supporting the retrieval and the discrimination of the temporal context of experienced events in retrospective time estimation paradigms, and the possible differential role of hippocampal subfields.

## Data Availability

The conditions of our ethics approval do not permit public archiving of the raw MRI data. The preprocessed MRI anonymous data are available at the corresponding author on reasonable request.
